# Prevalence of Ingested Fish Hooks in Freshwater Turtles from Five Rivers in the Southeastern United States

**DOI:** 10.1371/journal.pone.0091368

**Published:** 2014-03-12

**Authors:** David A. Steen, Brittney C. Hopkins, James U. Van Dyke, William A. Hopkins

**Affiliations:** 1 Department of Fish and Wildlife Conservation, Virginia Tech, Blacksburg, Virginia, United States of America; 2 School of Biological Sciences, A08 Heydon-Laurence Building, University of Sydney, Sydney, Australia; National University of Mongolia, Mongolia

## Abstract

Freshwater turtles may ingest baited fish hooks because many are opportunistic scavengers. Although the ingestion of fish hooks is known to be a source of mortality in multiple vertebrate groups, the prevalence of hook ingestion by freshwater turtles has not been well studied. We trapped turtles from five rivers in the southeastern United States and used radiographs to examine over 600 individuals of four species. Depending on the species, sex, and age class, 0–33% of turtles contained ingested fish hooks. For some species, larger turtles were more likely to contain a fish hook than smaller individuals. Freshwater turtle demography suggests that even small increases in adult mortality may lead to population declines. If our study areas are representative of other aquatic systems that receive fishing pressure, this work likely identifies a potential conflict between a widespread, common recreational activity (i.e., fishing) and an imperiled taxonomic group.

## Introduction

Recreational fishing is a widespread activity [1], [2], that poses threats to aquatic wildlife assemblages through the production of bycatch [3]. Bycatch may be a particularly important threat for populations of imperiled taxa, such as some turtles [4]. Several studies have described the capture of freshwater turtles in fish traps [5]–[8] and of estuarine turtles (i.e., diamondback terrapins, *Malaclemys terrapin* Schoepff 1793) in crab traps [9]. Because freshwater turtles are opportunistic scavengers and also take live prey, they are also likely vulnerable to capture with baited hooks set to catch fish [10]; in fact, they are targeted by commercial and recreational collectors via this same method [11].

Although freshwater turtles may ingest fish hooks [10], [12], which can negatively affect their health [13], there are few data to indicate whether fish-hook ingestion is of large-scale conservation concern. For example, fishing-gear related trauma is a commonly reported injury for reptiles admitted to wildlife rehabilitation centers [14], but these cases represent a biased sample that does not identify the proportion of free-ranging animals affected. However, fish hooks were found in three of 17 (∼18%) X-rayed female European pond turtles, *Emys orbicularis* Linnaeus 1758, from a heavily-fished series of ponds in France [15], suggesting significant proportions of turtles may be affected.

Hook ingestion causes elevated mortality rates in several taxa (e.g., sea turtles, fish, and birds) [16]–[18]. Given the highly imperiled status of freshwater turtles in general [4] and the suggested inability of their populations to persist when exposed to even low levels of adult mortality [19], [20], it is important to identify potential conflicts with widespread anthropogenic activities such as recreational fishing. To this end, we sampled freshwater turtles in five rivers in the Southeastern United States and used X-ray radiography [21] to quantify the proportion of animals that contained fish hooks while determining how the sex, size and species of an individual turtle might influence its relative vulnerability to fish hook ingestion.

## Materials and Methods

### Study Sites

The work described herein was done opportunistically as components of two larger and independent studies examining anthropogenic effects on the reproductive ecology of turtles in Tennessee and Virginia, U.S.A. In Tennessee, our study site was a continuous riverine habitat centered around Kingston that included the Emory (river km 0.0–5.5), Clinch (river km 0.0–7.0), and Tennessee Rivers (river km 914–922). The area is open to the public and accessible via numerous boat launches; common recreational uses include fishing, boating, and other water sports. We observed three primary fishing methods within the study area, bass fishing with artificial lures, fishing with live bait, and unattended lines with baited hooks attached to floats. Full Tennessee fishing regulations can be found elsewhere [22].

In Virginia, our study sites included the South and Middle Rivers around Waynesboro and Staunton. The land adjacent to our study areas on the South River is forested in the upper mountainous regions but at lower elevations the river runs through small-urbanized areas and private land that is used mainly for agriculture and livestock. Our study areas on the Middle River flow primarily through rural areas and are surrounded by privately owned farms. Although public access is limited on the Middle River, several public access areas are present on the South River, including popular swimming spots and areas that experience trout and bass fishing. A health advisory for fish consumption exists within several of our sampling areas along the South River but we frequently observed recreational fishing with artificial lures, baited hooks, and in some areas flyfishing. In addition, informal conversations with anglers suggested that fish advisories were not entirely effective at deterring people from catching and consuming turtles. Representative Virginia fishing regulations can be found elsewhere [23].

### Trapping

Turtles were captured in baited hoop traps (Memphis Net and Twine, Memphis, TN, USA). No turtles were harmed in this study; all individuals were released at their point of capture after processing. Standard morphological measurements were taken on turtles and they were sexed based on secondary sexual characteristics. We included four species in the current study: Eastern Musk Turtles (*Sternotherus odoratus* Latreille in Sonnini & Latreille 1801), Pond Sliders (*Trachemys scripta* Schoepff 1792), Spiny Softshells (*Apalone spinifera* LeSueur 1827), and Snapping Turtles (*Chelydra serpentina* Linneaus 1758). These species are generally considered common throughout their range but their life histories are representative of those of many other chelonian species that are uncommon and in some cases of great conservation concern. We considered *T. scripta* males and females as adult if they were >11 cm and >20 cm carapace length (CL), respectively, *S. odoratus* males and females as adult if they were >6 cm and >8 cm CL, respectively, *A. spinifera* males and females as adult if they were >130 g and >20 cm CL, respectively, and *C. serpentina* of either sex as adult if they were >20 cm CL [24].

In Tennessee, we X-rayed all female *S. odoratus*, *T*. *scripta*, *A. spinifera*, and *C. serpentina* known or suspected to be as gravid based on physical palpation between 5 May and 25 July 2012. Between 16 June and 25 July we X-rayed additional turtles, including males, as time allowed. In Virginia, we collected *C. serpentina* from April-July in 2010 and 2011, as described in Hopkins et al. [25], and gathered data only on female turtles. The turtles we decided to X-ray are not necessarily representative of the relative abundances of the various species or of the age and sex distributions present within the population.

### X-ray

None of the turtles we X-rayed displayed any visible evidence of hook ingestion (i.e., there were no externally visible hooks and/or fishing line). In Tennessee, we used an Ecoray Ultralight 9020 HF set at 70 kV and 4.00 mA to X-ray turtles. In Virginia, turtles were X-rayed by technicians at the Wildlife Center of Virginia with a Summit Innovet and settings were adjusted as required. For both sites, we recorded the presence/absence of fish hooks in X-rays. We also attempted to identify the type of fish hook (i.e., J, circle, or treble) based on their shape, but we were unable to reliably differentiate J hooks from circle hooks because hooks were lodged in turtles at varying angles.

### Ethics Statement

Capture and handling of turtles was approved by the animal care and use committee at Virginia Polytechnic and State University (IACUC # 09-080-FIW, 10-055-FIW, 11-044-FIW, and 12-056-FIW) and appropriate state collection permits were obtained (Virginia # 035981; Tennessee # TN3610).

### Statistical Analysis

We used separate logistic regressions to analyze the presence and absence of fish hooks in turtles from Tennessee and Virginia. In Tennessee turtles, we examined the effects of species, carapace length, and sex. In Virginia turtles, we examined only the effect of carapace length because all X-rayed turtles were female *C. serpentina*. We modeled logistic regressions using PROC GLIMMIX in SAS 9.3 (SAS Institute, Cary NC). We log-transformed carapace length in the analysis for Virginia turtles because it was found to improve the fit of the model, as judged by reduced AIC values. The interaction between carapace length and species was significant in the Tennessee model, so we used post-hoc logistic regressions to examine the effect of carapace length on hook presence/absence within each species. Because there were low absolute numbers of turtles with ingested fish hooks, we ran power analyses (PROC POWER) on the variance outputs from the analysis of the Tennessee turtles to determine whether our sample sizes were large enough to avoid committing type II errors (i.e., failing to reject false null hypotheses).

## Results

In Tennessee, we X-rayed a total of 84 *A. spinifera* (25 adult males, 50 adult females, and 9 juveniles), 20 *C. serpentina* (10 adult males, 9 adult females, and 1 juvenile), 92 *S. odoratus* (24 adult males and 68 adult females), and 242 *T. scripta* (115 adult males, 106 adult females, and 21 juveniles). No hooks were detected in *S. odoratus*. Of species that contained hooks, the proportion of adult males and females with ingested hooks ranged from 3.5–10% and 6–33%, respectively (Table 1). In Virginia, we X-rayed a total of 170 *C. serpentina*. Of the 168 adult females from this sample, 6 (3.6%) contained ingested hooks (Table 1).

**Table 1:** Total turtles X-rayed and proportion containing fish hooks from the Clinch, Emory, and Tennessee Rivers, Tennessee, and South and Middle Rivers, Virginia.

Location	Species	Life Stage	Number X-rayed	Total Hooked	Proportion hooked
Tennessee	*Sternotherus odoratus*				
		Adult males	24	0	0.00
		Adult females	68	0	0.00
	*Chelydra serpentina*				
		Adult males	10	1	10.00
		Adult females	9	3	33.33
		Juveniles	1	0	0.00
	*Trachemys scripta*				
		Adult males	115	4	3.48
		Adult females	106	9	8.49
		Juveniles	21	0	0.00
	*Apalone spinifera*				
		Adult males	25	1	4.00
		Adult females	50	3	6.00
		Juveniles	9	0	0.00
Virginia	*Chelydra serpentina*				
		Adult females	168	6	3.57
		Juveniles	2	0	0.00

**Figure 1:**
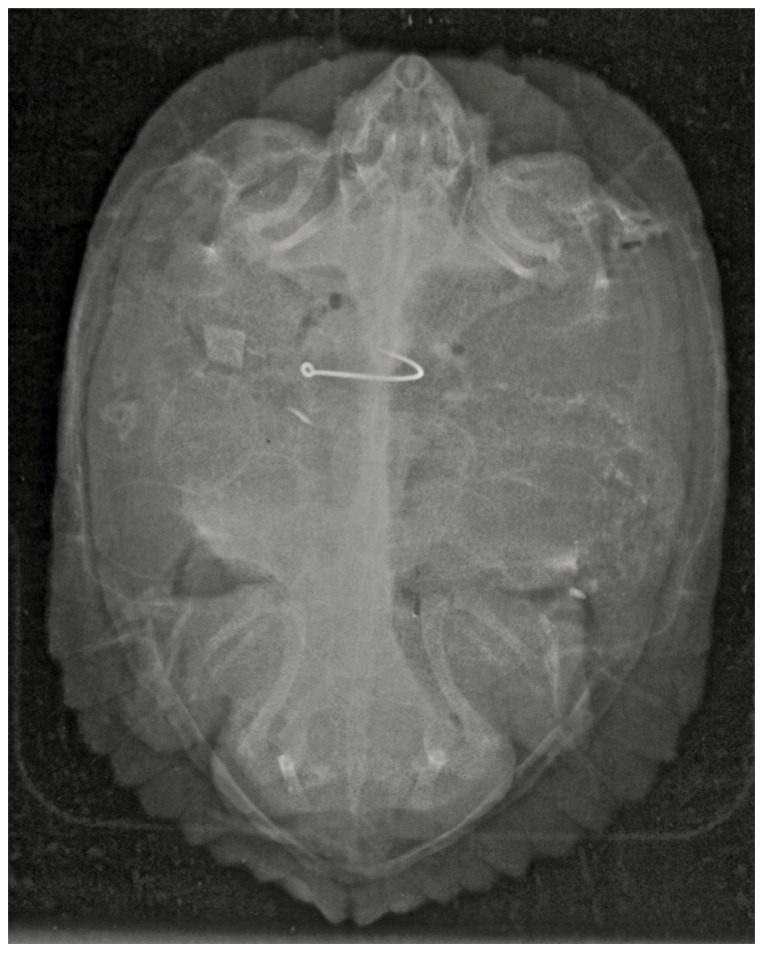
X-ray of a gravid Pond Slider (*Trachemys scripta*) captured in Tennessee containing a fish hook. Image has been enhanced to improve hook visibility.

**Figure 2:**
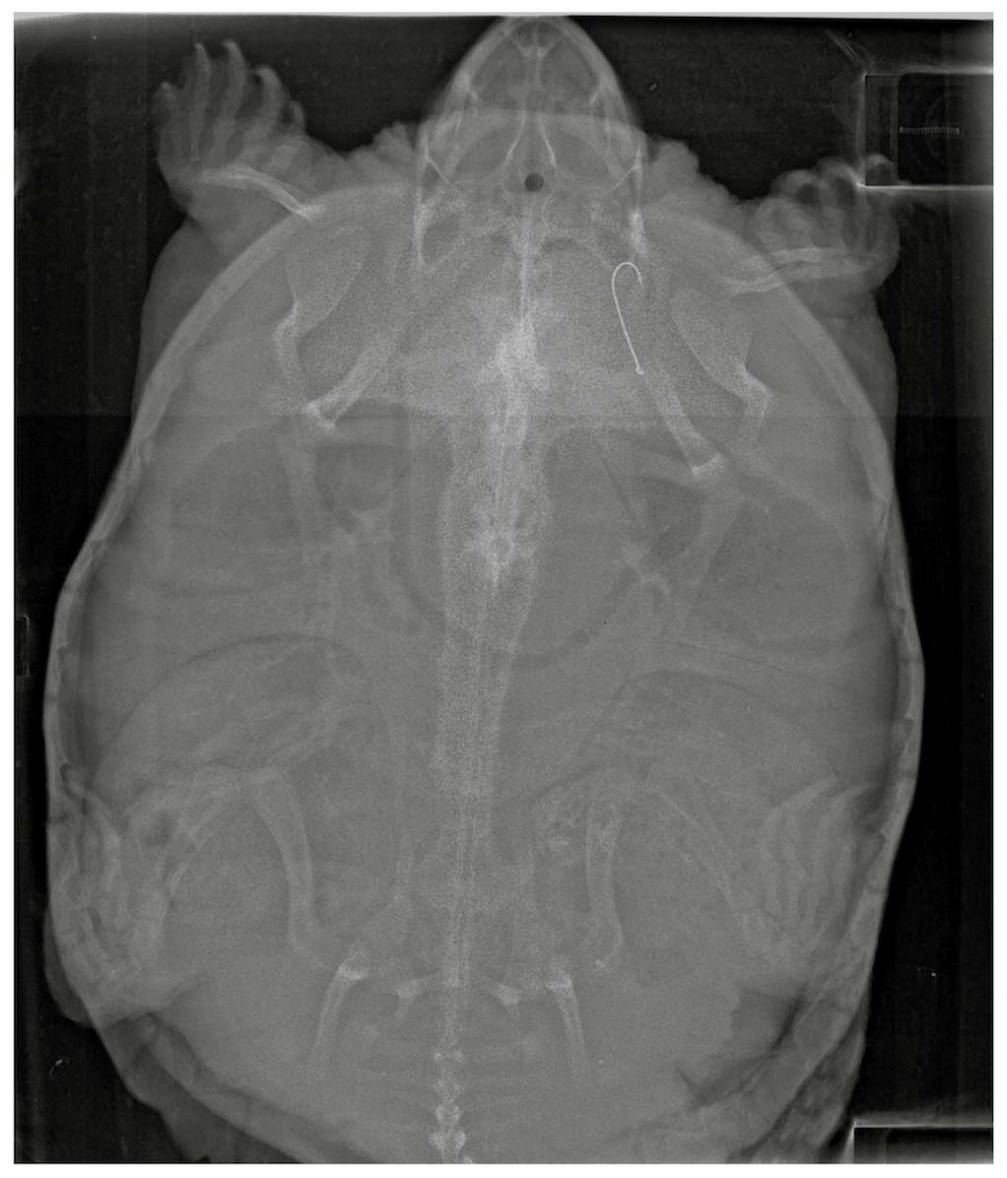
X-ray of a Snapping Turtle (*Chelydra serpentina*) captured in Tennessee containing a fish hook. Image has been enhanced to improve hook visibility.

**Figure 3:**
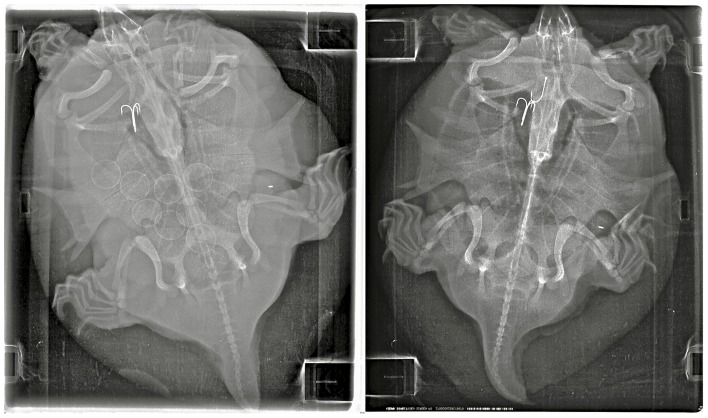
A Spiny Softshell (*Apalone spinifera*) first captured on 4 June 2012 (left) while gravid with eight eggs and containing one treble fish hook and again captured on 27 July 2012 (right) with an additional fish hook. A Passive Integrated Transponder (PIT tag) is visible in both X-rays. Image has been enhanced to improve hook visibility.

**Figure 4:**
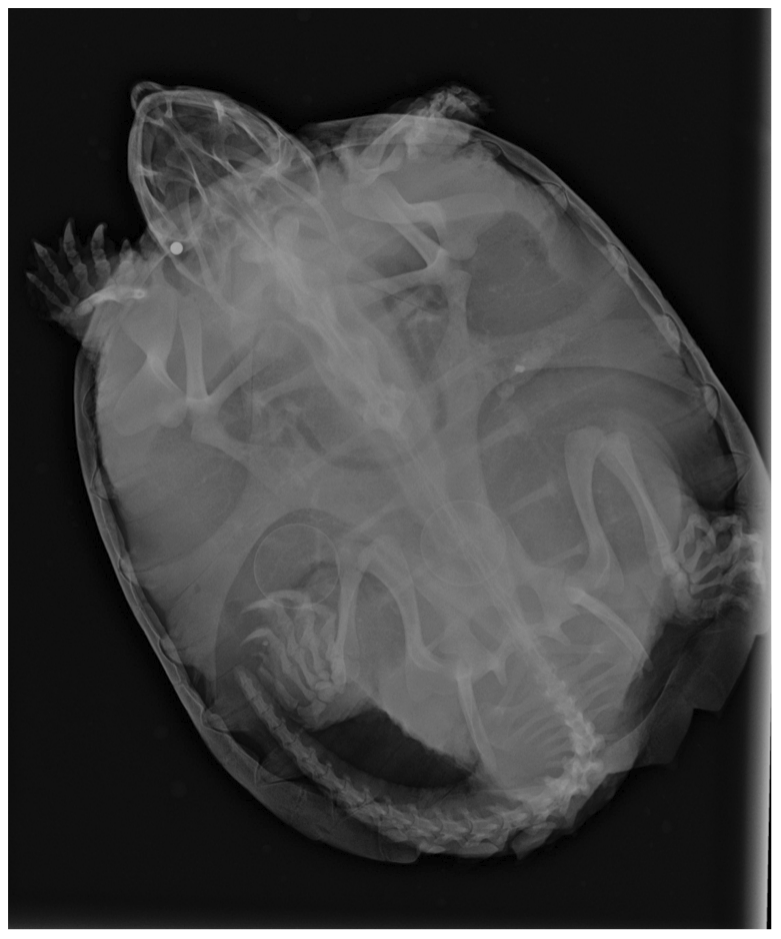
X-ray of a gravid Snapping Turtle (*Chelydra serpentina*) captured in Virginia containing a metal pellet in its jaw.

In all but one instance, ingested hooks appeared to be J or circle hooks (as depicted in Figure 1 and 2) and were present in the esophagus or abdomen. A gravid *A. spinifera* from Tennessee captured on 4 June 2012 contained a treble hook. This individual was re-captured on 27 July 2012 and X-rayed again as part of the independent reproductive ecology study; we noticed she was no longer gravid and contained a J-hook in addition to the treble hook, which had not appreciably shifted its location or orientation (Figure 3). A female *T. scripta* from Tennessee and a female *C. serpentina* from Virginia both contained two hooks. Another *T. scripta* from Tennessee contained a hook and a barrel swivel. We also observed small (<10 mm in diameter) metal pellets in the jaw region (Figure 4) of two *C. serpentina* from Virginia, including the individual that had swallowed two hooks (not pictured). We believe these pellets are associated with the recreational shooting of turtles (e.g., “plinking”) [24].

For Tennessee turtles, hook presence/absence was significantly affected by the interaction between species and carapace length (Table 2). Post hoc within-species analyses investigating only the effects of carapace length showed that large *T. scripta* were more likely to contain hooks than were small *T. scripta* (*F*_1, 235_ = 6.11, *P* = 0.014; Figure S1 A). In contrast, carapace length did not affect hook presence/absence in either *A. spinifera* (*F*_1, 80_ = 0.05, *P* = 0.825; Figure S1 B) or *C. serpentina* (*F*_1, 16_ = 6.11, *P* = 0.271; Figure S1 C). We did not examine the effect of carapace length on hook presence/absence in *S. odoratus* alone because no *S. odoratus* contained hooks. For Virginia female *C. serpentina*, hook presence/absence was significantly affected by carapace length (*F*_1, 237_ = 6.65, *P* = 0.011; Figure S1 D); larger turtles were more likely to contain ingested hooks than were smaller turtles. Power analyses confirmed that our sample sizes were sufficient to avoid committing type II errors; in all comparisons, the probability of rejecting false null hypotheses was greater than 99.9%.

**Table 2:** Results of mixed-model logistic regression analyses of the presence and absence of fish hooks in turtles from the Clinch, Emory and Tennessee Rivers, Tennessee.

Source	Numerator df	Denominator df	F	P
Carapace Length	1	419	0.12	0.728
Sex	1	419	0.02	0.899
Species	3	419	1.43	0.233
Carapace Length X Species	3	419	8.32	<0.001*
Carapace Length X Sex	1	419	1.83	0.177
Sex X Species	3	419	0.88	0.451
Carapace Length X Sex X Species	2	419	1.08	0.342

Asterisks indicate factors significant at α = 0.05.

## Discussion

Recreational activities have the potential to negatively influence freshwater turtles, a group that faces a myriad of additional conservation threats [26]–[28] that may act in concert to imperil their populations. Here, we add to the body of knowledge regarding freshwater turtle conservation by reporting the proportions of freshwater turtles captured at our study sites that contained ingested fish hooks. Given the injuries associated with hook ingestion in other taxa (e.g., [16]–[18]), our data suggest that recreational fishing is a potential anthropogenic threat for this imperiled group. However, our study likely underestimates the total proportions of the freshwater turtle populations that ingested fishing tackle because the turtles we identified as containing hooks are only those individuals that swallowed hooks, escaped or were released by anglers, and survived the time from being hooked until time of capture in our study without expelling the hook. In addition, in areas where turtles are intensively harvested (recreationally or commercially) via baited hooks (e.g., [11]) or where fishing pressure is higher than in our study sites, the proportions of turtles with ingested hooks could be considerably higher than we observed.

The likelihood of a hook being ingested by a sea turtle may be influenced by the species, the size of the animal, and the type and size of the hook [29]. In Tennessee *T. scripta* and Virginia *C. serpentina*, we demonstrated that relatively large turtles are more likely to contain an ingested hook than smaller individuals. Potential reasons for size effects on hook presence/absence for these turtles include gape limitations, the possibility that larger, older turtles have had a longer period of time to accumulate fishing gear, and/or that small turtles die relatively quickly after ingesting hooks, making them less available for capture (e.g., [30], [31]). Adult females represent the demographic class that is most important for population persistence [19], [20]; because adult females grow larger than males in most freshwater turtle species (but notably, not *C. serpentina*), this group may be disproportionately vulnerable to ingesting fish hooks, as they are to boat propeller collisions and road mortality [28], [32], [33].

We did not observe size effects in Tennessee *A. spinifera* or *C. serpentina*, suggesting that large turtles may not be more vulnerable to hook ingestion than small turtles in all species or populations. Given both the results of our power analyses and considerable ranges in body sizes for *A. spinifera* (CL mean = 25.8 cm, SE = 0.9 range = 13.0–39.7 cm) and *C. serpentina* (CL mean = 28.2 cm, SE = 1.1, range = 15.2–35.4 cm) as well as *T. scripta* (CL mean = 19.7, SE = 0.2, range = 11.5–25.7 cm), we cannot clearly attribute this inconsistency to an inability to detect size effects in *A. spinifera* and *C. serpentina*, if they existed. However, larger sample sizes that included more small individuals may be useful for further examining this potential. Although we did not observe any *S. odoratus* with ingested hooks, elsewhere they are frequently hooked in the mouth by anglers using baited hooks (DAS personal observation). We suggest that this species is likely too small (8–12 cm in our study) to swallow typical fish hooks and hooks in the mouth may be removed relatively easily by anglers. In species that grow to large sizes, such as *A. spinifera*, *C. serpentina*, and *T. scripta*, small individuals may also be too small to ingest hooks, but our dataset included few individuals smaller than 11 cm in carapace length. We lack information regarding how hook ingestion affects the physiology and health of freshwater turtles; this is not surprising given the limited studies of the subject pertaining to any taxa (e.g., [34]–[36]). However, ingestion of fish hooks leads to increased mortality rates in birds, fish, mammals, and sea turtles [18], [30], [37], [38]. Sea turtles hooked in the esophagus may experience anything from no observed effects to infections causing systemic septicemia [39]. The lining of a sea turtle stomach is thinner than that of their esophagus and hooks in this region are more likely to result in punctures and coelomitis; if this occurs, mortality is often immediate [36], [38]–[39]. For deeply-hooked fish, survival rates are higher when no attempts are made to remove the hook [16]; the same may be true for turtles [36]. However, fish hook ingestion ultimately increases fish mortality regardless of whether hooks are removed [16]. Our knowledge of turtle demography, which includes low annual recruitment and delayed sexual maturity, suggests even small amounts of adult mortality (2–5%) above natural levels may lead to population declines [19], [20], [40].

Collaboration between researchers and commercial fishing operations has resulted in a relatively large body of knowledge regarding the prevalence of fish hook ingestion by sea turtles (e.g., [41]–[45]). This information has spurred and informed conservation recommendations and actions for that group [45]–[48]. However, fish-hook ingestion has not been thoroughly investigated as a conservation threat for freshwater turtles [49]. More research on the topic is needed to generate a better understanding of this conservation threat, including factors that influence the probability of hook ingestion and its consequences for the health and fitness of individual turtles. In the meantime, land managers, policy makers, and anglers should consider that recreational fishing might be affecting sensitive populations of freshwater turtles.

## Supporting Information

Figure S1 A:The relationship between length and mass for Pond Sliders (*Trachemys scripta*) captured in Tennessee found with or without ingested hooks. B: The relationship between length and mass for Spiny Softshells (*Apalone spinifera*) captured in Tennessee found with or without ingested hooks. C: The relationship between length and mass for Snapping Turtles (*Chelydra serpentina*) captured in Tennessee found with or without ingested hooks. D: The relationship between length and mass for Snapping Turtles (*Chelydra serpentina*) captured in Virginia found with or without ingested hooks.
(PDF)
