# Supplementary figures and images for: Prevalence of Ingested Fish Hooks in Freshwater Turtles from Five Rivers in the Southeastern United States
Source: PLoS One. 2014 Mar 12;9(3):e91368. doi: 10.1371/journal.pone.0091368 (PMC3951344; doi:10.1371/journal.pone.0091368)

A) *Trachemys scripta*

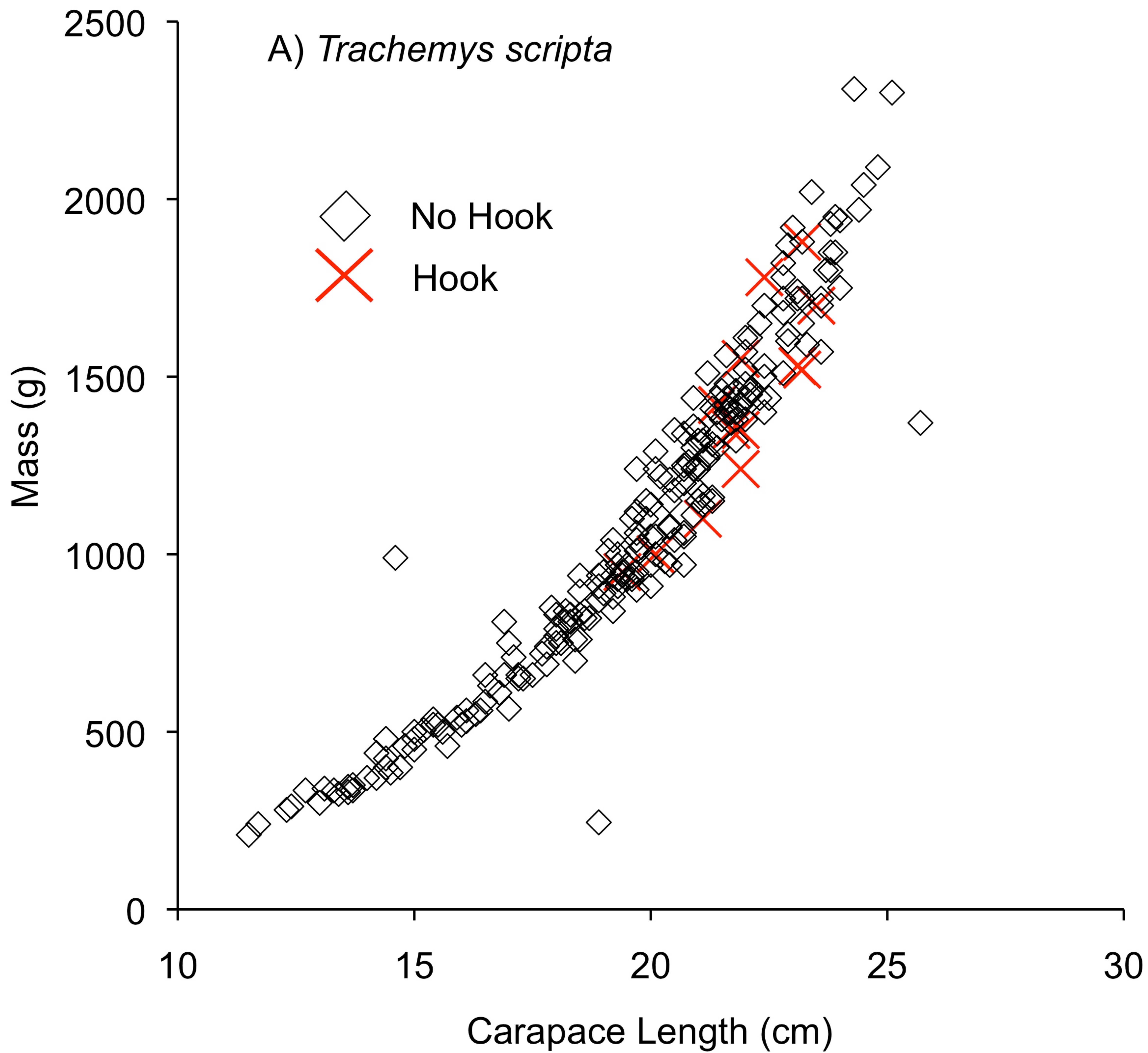

B) *Apalone spinifera*

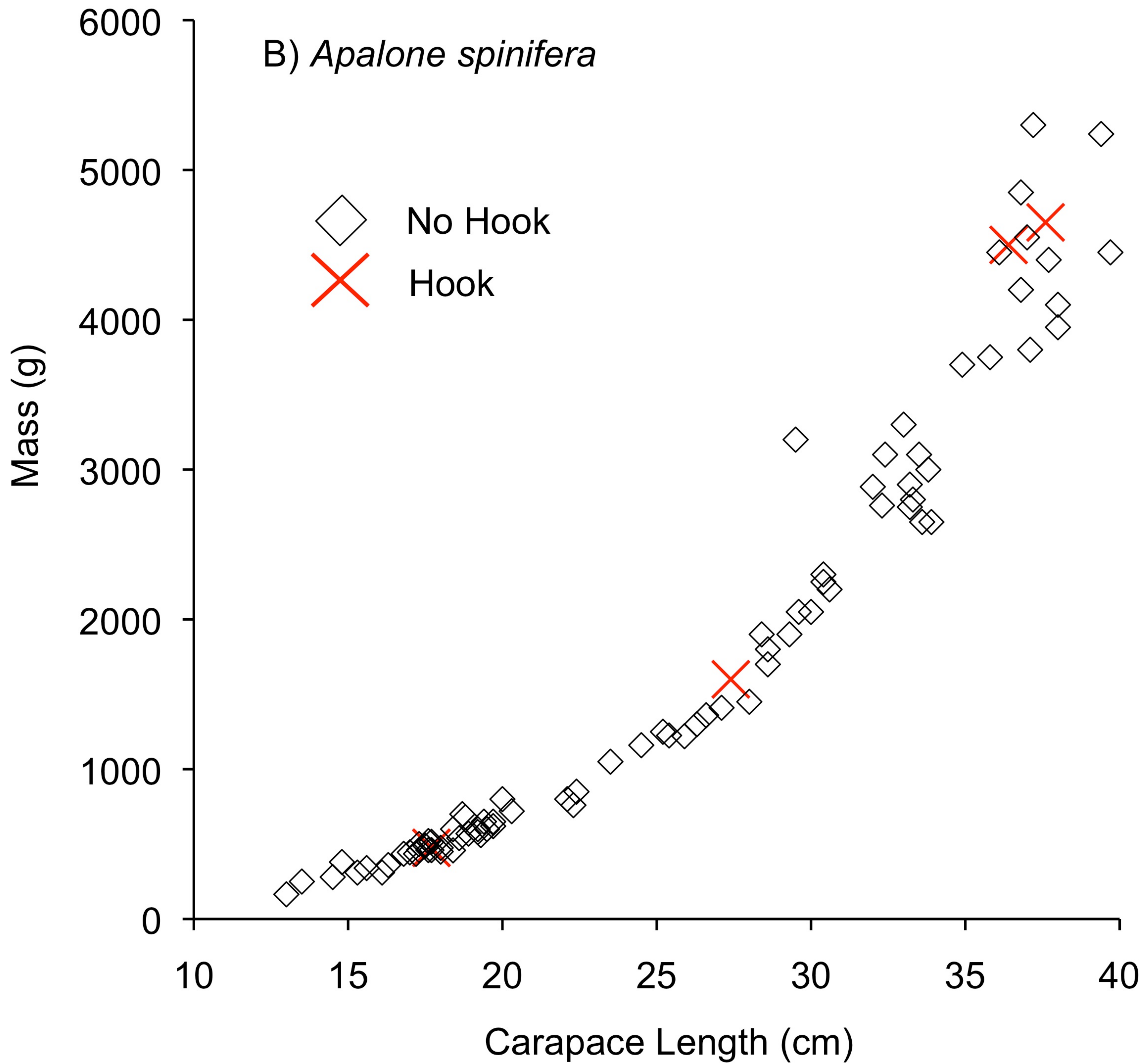

C) *Chelydra serpentina* (TN)

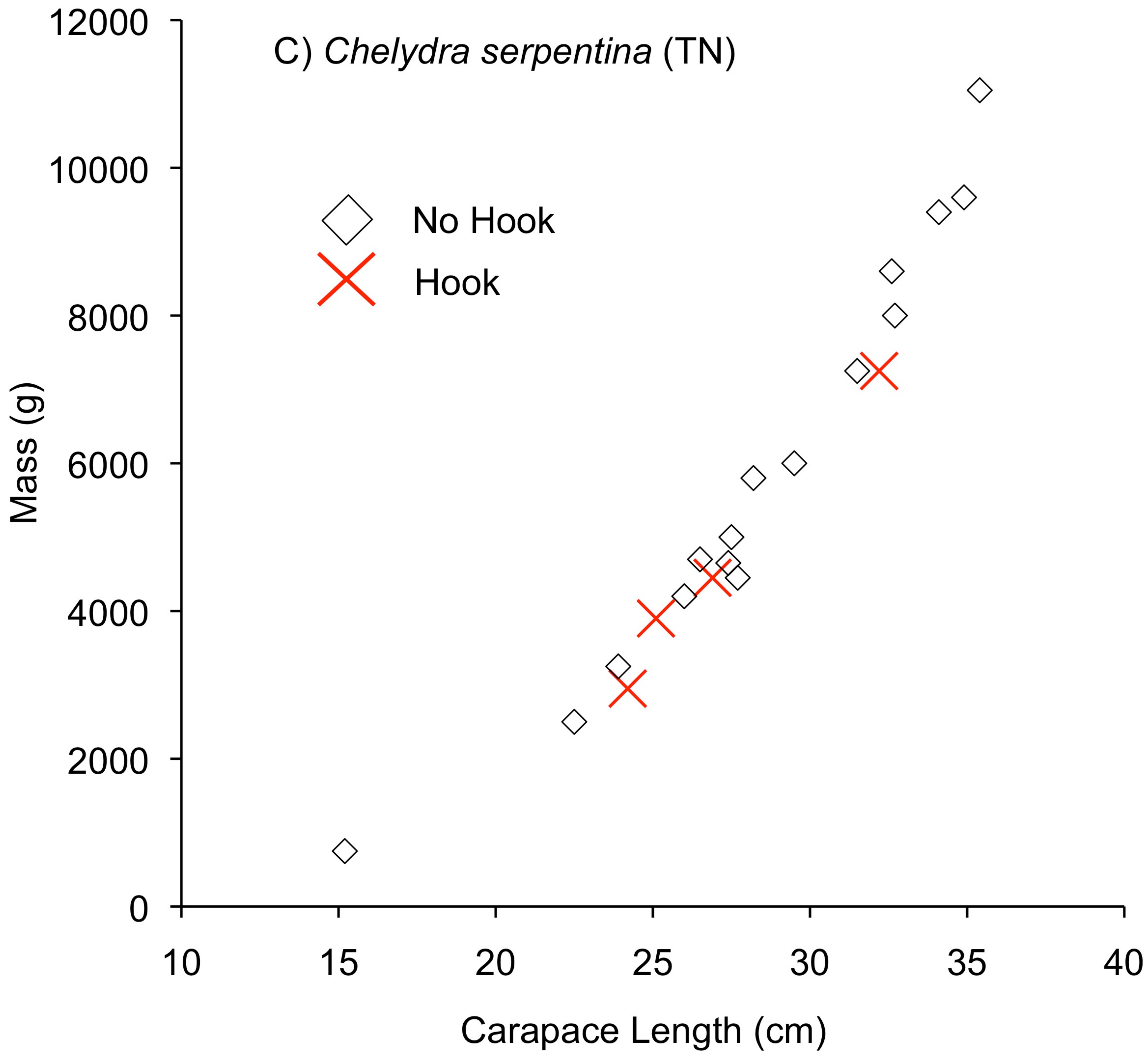

D) *Chelydra serpentina* (VA)

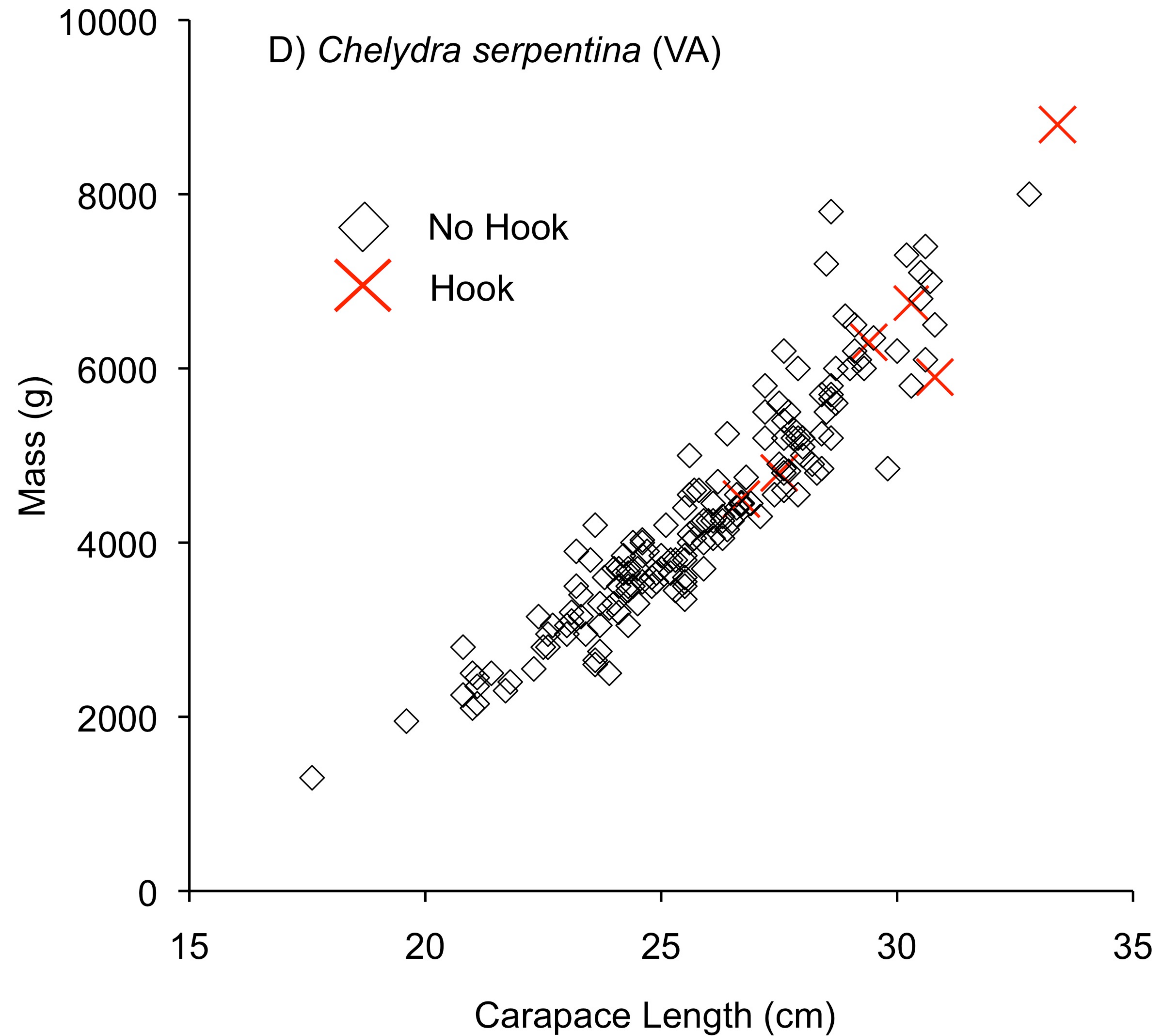

Supplement: Figure S1 — A:The relationship between length and mass for Pond Sliders (Trachemys scripta) captured in Tennessee found with or without ingested hooks. B: The relationship between length and mass for Spiny Softshells (Apalone spinifera) captured in Tennessee found with or without ingested hooks. C: The relationship between length and mass for Snapping Turtles (Chelydra serpentina) captured in Tennessee found with or without ingested hooks. D: The relationship between length and mass for Snapping Turtles (Chelydra serpentina) captured in Virginia found with or without ingested hooks. (PDF) [file pone.0091368.s001.pdf]
